# Respiratory Pathogens and Clinical Characteristics of Asthma Exacerbations in Hospitalized Children at a Tertiary Hospital in Türkiye

**DOI:** 10.3390/children13070853

**Published:** 2026-06-25

**Authors:** Enes Çelik, Hande Yüksel Bulut

**Affiliations:** Division of Pediatric Allergy and Immunology, Department of Pediatrics, Ankara Atatürk Sanatorium Training and Research Hospital, Ankara 06290, Türkiye; handeyuksel_88@hotmail.com

**Keywords:** asthma exacerbation, children, rhinovirus, respiratory syncytial virus, respiratory pathogens, aeroallergen sensitization, hospitalization

## Abstract

**Highlights:**

**What are the main findings?**

- Rhinovirus was the predominant respiratory pathogen among children hospitalized for asthma exacerbations and was associated with atopic features.
- Respiratory syncytial virus was detected mainly in younger children and was associated with greater severity only in unadjusted comparisons.

**What are the implications of the main findings?**

- Pathogen-specific clinical patterns may help clinicians interpret respiratory pathogen detections during asthma exacerbations.
- Younger age and moderate-to-severe asthma should be considered important risk indicators for severe exacerbation.

**Abstract:**

**Background/Objectives**: Respiratory pathogens are frequently detected during asthma exacerbations in children. This study aimed to evaluate the distribution of respiratory pathogens, associated clinical features, and factors related to severe exacerbation in children hospitalized for asthma exacerbations during a two-year period. **Methods**: This retrospective observational study included children aged 1–18 years hospitalized for asthma exacerbation between April 2023 and April 2025. Demographic, clinical, laboratory, and pathogen data were retrospectively obtained from medical records and analyzed. **Results**: A total of 312 children were included; 135 patients (43.3%) were female, and the median age was 5.70 years (IQR, 3.42–8.79). A respiratory pathogen was detected in 235 patients (75.3%). Among patients with single infections (*n* = 203), rhinovirus (RV) was the most common pathogen (*n* = 130), followed by respiratory syncytial virus (RSV) (*n* = 18). Compared with RSV infection, RV infection was associated with higher frequencies of allergic rhinitis and aeroallergen sensitization, as well as higher neutrophil and eosinophil counts and higher total IgE levels. RV was detected throughout the year, peaking in autumn, whereas RSV occurred predominantly in winter. RSV infection was observed in younger children and was associated with more frequent severe exacerbations in unadjusted comparisons; however, only younger age and moderate-to-severe asthma remained independently associated with severe exacerbation in multivariable analysis. **Conclusions**: Respiratory pathogens were detected in most children hospitalized for asthma exacerbation, with RV being the predominant pathogen. Younger age and moderate-to-severe asthma were the main factors associated with severe exacerbation.

## 1. Introduction

Asthma is the most prevalent chronic respiratory disease in children, with global childhood prevalence estimated at approximately 10% [1,2]. It is a heterogeneous disorder characterized by various phenotypes and endotypes, with respiratory infections commonly associated with exacerbations [3,4,5]. Rhinovirus (RV) and respiratory syncytial virus (RSV) are among the most frequently detected pathogens during asthma exacerbations, although other viruses, including influenza virus, parainfluenza virus, coronavirus, metapneumovirus, and adenovirus, may also be detected [6,7,8]. These viruses may also differ in their clinical associations, as RSV-related wheezing has been reported to be more common in infants and non-atopic children, whereas RV-related wheezing is more closely linked to pre-existing atopy [9,10].

In a recent study of children with asthma exacerbations presenting to a pediatric emergency department, at least one respiratory virus was detected in 75.0% of those who underwent respiratory virus polymerase chain reaction (PCR) testing, with RV being the most frequently identified virus [11]. Moreover, a population-based study of children younger than 15 years hospitalized with asthma reported that the proportion of RSV infection increased from 1.44% in 2016 to 7.4% in 2022 [12].

Viral infections play a significant role, particularly during times when children return to school following summer and spring breaks [13]. RV circulates year-round, with peak prevalence in early autumn and late spring, whereas RSV is typically observed during the winter season [14]. Periods characterized by heightened circulation of respiratory viruses have been linked to worsening asthma symptoms and a higher likelihood of loss of disease control [15].

RV is highly prevalent among respiratory viruses. However, most RV infections in children with asthma do not result in exacerbations [14,16]. Therefore, host-related factors such as allergic sensitization and airway inflammation may influence susceptibility to RV-associated exacerbations [17].

There is a need for studies evaluating respiratory pathogens in pediatric asthma exacerbations across multiple seasons. The aim of this study was to evaluate the distribution of respiratory pathogens, associated clinical features, and factors related to severe exacerbation in children hospitalized for asthma exacerbations during a two-year period.

## 2. Materials and Methods

### 2.1. Study Design and Population

This retrospective observational study was conducted at the Division of Pediatric Allergy and Immunology, Ankara Atatürk Sanatorium Training and Research Hospital, Ankara, Türkiye, between 1 April 2023, and 1 April 2025. Participants were eligible if they were aged 1–18 years, had a respiratory pathogen panel performed, and had a diagnosis of asthma either previously established or confirmed during hospitalization and subsequent outpatient follow-up in our pediatric allergy and immunology clinic. For patients with more than one hospitalization during the study period, only the first hospitalization was included in the analysis. Children were not included if they had been evaluated only during hospitalization without documented asthma follow-up before or after admission at our pediatric allergy and immunology clinic, were hospitalized for a primary condition other than asthma exacerbation, such as bronchiolitis or pneumonia, or had immunodeficiency, congenital heart disease, prematurity <32 weeks of gestation, bronchopulmonary dysplasia, primary ciliary dyskinesia, cystic fibrosis, other chronic conditions, or incomplete data. Patients’ demographic and clinical characteristics and laboratory findings were obtained from electronic medical records. White blood cell (WBC) count, differential leukocyte counts, and C-reactive protein (CRP) levels were routinely measured using blood samples collected within the first hour after presentation to our hospital.

Asthma was diagnosed according to the Global Initiative for Asthma (GINA) criteria, based on a history of variable respiratory symptoms (wheeze, shortness of breath, chest tightness, and cough) and the presence of variable expiratory airflow limitation [18]. For children aged ≤5 years, asthma was diagnosed by pediatric allergy and immunology specialists based on recurrent wheezing episodes or at least one acute wheezing episode with asthma-like symptoms between episodes, absence of an alternative diagnosis, supportive clinical features including allergic disease, allergen sensitization or asthma in first-degree relatives, and clinical improvement with asthma treatment when applicable [18]. Asthma exacerbations were defined according to the GINA recommendations available during the study period as a worsening of symptoms and lung function beyond the patient’s baseline condition. Underlying asthma severity was classified according to the same recommendations based on the level of treatment required to achieve asthma control and was categorized as mild or moderate-to-severe asthma for analysis [18]. The severity of acute asthma exacerbation was assessed using the Pediatric Asthma Severity Score (PASS), which includes three clinical components—wheezing, work of breathing, and prolongation of expiration—each scored from 0 to 2, yielding a total score ranging from 0 to 6. For descriptive purposes, PASSs were categorized as mild (0–2), moderate (3–4), and severe (5–6) [19].

### 2.2. Pathogen Detection

Respiratory pathogen panel testing was routinely performed in children hospitalized for asthma exacerbation at our institution. Nasopharyngeal swab samples were obtained within the first 2 h of admission by experienced clinicians and processed in accordance with the manufacturer’s instructions. Pathogen detection was performed using real-time polymerase chain reaction (RT-PCR) with the Bosphore Respiratory Pathogens Kit (Anatolia Geneworks, Istanbul, Türkiye), and was defined as laboratory identification of viral or bacterial targets by RT-PCR from nasopharyngeal samples. Coinfection was defined as the detection of more than one pathogen by RT-PCR, regardless of whether the detected organisms were viral or bacterial. The following respiratory pathogens were analyzed: influenza A (H1, H1N1/2009, and H3), influenza B, RSV A/B, adenovirus, enterovirus, parainfluenza viruses (types 1–4), human metapneumovirus, bocavirus, RV, human coronaviruses (NL63, OC43, 229E, and HKU1), parechovirus, *Mycoplasma pneumoniae*, *Chlamydia pneumoniae*, *Legionella pneumophila*, *Haemophilus influenzae*, *Streptococcus pneumoniae*, and *Bordetella pertussis*. SARS-CoV-2 was not included in the respiratory pathogen RT-PCR panel. However, according to institutional practice, rapid antigen testing was routinely performed in patients with clinical suspicion of COVID-19, and no SARS-CoV-2-positive cases were identified among the study population.

### 2.3. Allergy Testing

Allergic evaluation was routinely performed during outpatient follow-up at our pediatric allergy and immunology clinic, rather than during the acute asthma exacerbation, using skin prick testing (SPT) and/or measurement of allergen-specific IgE levels according to the clinical status of each child. Because all included children were followed in our pediatric allergy and immunology clinic for asthma management, aeroallergen sensitization data were available for all patients included in the study. Total IgE levels were obtained from outpatient allergy follow-up records. SPT was performed with standardized allergen extracts (Lofarma, Milan, Italy). The aeroallergen panel included house dust mites, grass pollen mix, cereal pollen mix, tree pollen mix, *Cupressus*, *Plantago*, *Artemisia*, *Ambrosia*, *Chenopodium*, *Parietaria*, *Alternaria*, *Aspergillus*, as well as cat, dog, and cockroach allergens. A positive SPT result was defined as a wheal diameter ≥ 3 mm greater than that of the negative control. Allergen-specific IgE levels were measured for house dust mite, grass pollen mix, mold mix, and animal dander mix, with concentrations ≥ 0.35 kU/L considered indicative of sensitization. Total and specific IgE levels were measured using a chemiluminescent immunoassay system (IMMULITE 2000 XPI, Siemens, Erlangen, Germany).

### 2.4. Statistical Analysis

Statistical analyses were performed using IBM SPSS Statistics (version 22.0; IBM Corp., Armonk, NY, USA). The normality of data distribution was assessed using the Kolmogorov–Smirnov and Shapiro–Wilk tests. As none of the continuous variables followed a normal distribution, all continuous variables are presented as median (interquartile range, IQR). The Mann–Whitney U test was used to compare non-normally distributed variables. Categorical variables were compared using Pearson’s chi-square test or Fisher’s exact test, as appropriate. Logistic regression analysis was performed to identify factors associated with severe exacerbations. Candidate variables for the multivariable model were identified using a univariate screening threshold of *p* < 0.20, combined with clinical plausibility. Multicollinearity among the candidate variables was assessed using variance inflation factors (VIF) and no relevant multicollinearity was detected. Because only 24 children experienced a severe exacerbation, corresponding to an events-per-variable ratio of 8 for the final three-variable model, a Firth penalized logistic regression approach was used to reduce small-sample bias and to provide stable estimates for rare exposures. This analysis was performed in R (version 4.5.2; R Foundation for Statistical Computing, Vienna, Austria) using the logistf package (version 1.26.1). Odds ratios (OR) with 95% profile-penalized-likelihood confidence intervals (CI) were calculated. Overall model significance was evaluated using the likelihood ratio test, and model discrimination was assessed using the area under the receiver operating characteristic curve (AUC). Odds ratios with 95% CIs were presented graphically using GraphPad Prism (version 11.0.2; GraphPad Software, Boston, MA, USA). Analyses were performed using available data for each variable, and no imputation procedures were applied. A *p* value < 0.05 was considered statistically significant.

The study was conducted in accordance with the principles of the Declaration of Helsinki and received approval from the Scientific Research Ethics Committee of Ankara Atatürk Sanatorium Training and Research Hospital (Approval No: 2024-BÇEK/300). The study was approved as a retrospective review using anonymized medical records. No patient contact or intervention was involved, and the requirement for informed consent was waived by the Ethics Committee.

## 3. Results

**Figure 1:**
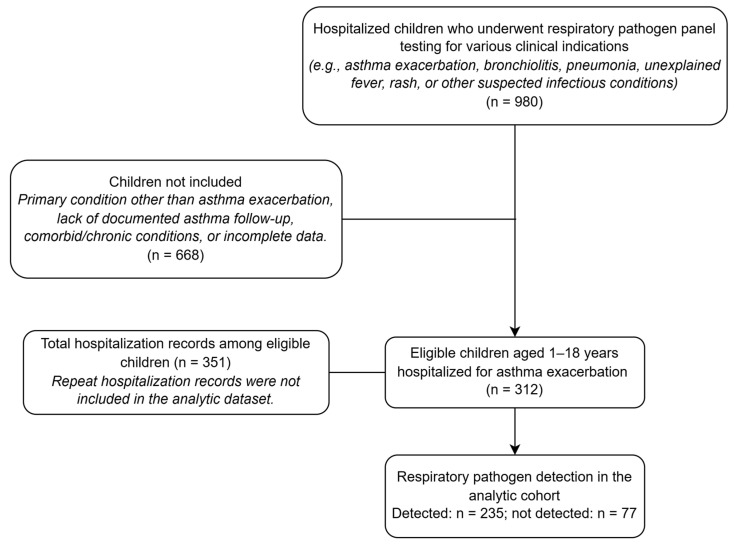
Flow diagram of patient selection and analytic cohort structure.

During the study period, 980 hospitalized children underwent respiratory pathogen panel testing for various clinical indications, including asthma exacerbation, bronchiolitis, pneumonia, unexplained fever, rash, and other suspected infectious conditions. After review of medical records, 312 children aged 1–18 years who were hospitalized for asthma exacerbation met the eligibility criteria and were included in the study. These children accounted for 351 hospitalization records during the study period; to avoid non-independence of repeated observations, only the first hospitalization of each child was included in the analysis. The full analytic cohort therefore consisted of 312 children (Figure 1). The comparison according to pathogen detection status included all 312 children, comprising 235 pathogen-positive and 77 pathogen-negative children (Table 1). Subgroup analyses were then performed using the relevant comparison groups: children with single RV (*n* = 130) or RSV (*n* = 18) detection for the RV–RSV comparison (Table 2), and children with single pathogen detection (*n* = 203) or coinfection (*n* = 32) among pathogen-positive children (Table 3).

Overall, 135 patients (43.3%) were female, and the median age was 5.70 years (IQR, 3.42–8.79). Preterm birth was present in 32 patients (10.3%), a family history of atopy in 89 (28.5%), exposure to cigarette smoking in 198 (63.5%), and parental consanguinity in 39 (12.5%). In terms of asthma severity, 168 patients (53.8%) had mild asthma, while 144 (46.2%) had moderate-to-severe asthma. Allergic rhinitis was the most frequent comorbid allergic disease, observed in 143 patients (45.8%), followed by atopic dermatitis in 57 patients (18.3%). The median length of hospital stay was 5 days (IQR, 4–6). Hospitalizations were most common in autumn (*n* = 127, 40.7%). Based on the PASS classification, 288 patients (92.3%) had mild-to-moderate exacerbations and 24 (7.7%) had severe exacerbations (Table 1).

A pathogen was detected in 235 of the 312 patients (75.3%), whereas no pathogen was identified in 77 patients (24.7%) (Table 1). Patients with pathogen positivity were significantly younger than those without an identified pathogen (median age, 5.50 [IQR, 3.14–8.43] vs. 6.87 [IQR, 3.90–9.90] years; *p* = 0.018).

Among the 235 patients with an identified pathogen, 203 (86.4%) had a single infection. In this single-infection subgroup, RV was the most frequently detected pathogen (*n* = 130), followed by respiratory syncytial virus (RSV) (*n* = 18). A comparison of patients with single RV and single RSV infections is presented in Table 2. Patients with RSV infection were younger than those with RV infection (median age, 3.7 [IQR, 1.9–5.5] vs. 5.8 [IQR, 3.6–9.7] years; *p* = 0.006). A higher proportion of patients with RSV infection were aged ≤5 years compared to those with RV infection (88.9% vs. 54.6%; *p* = 0.010). Mild asthma was more common among patients with RSV infection than those with RV infection (72.2% vs. 46.9%; *p* = 0.044). Despite this, severe attacks were more frequent in patients with RSV infection than in those with RV infection (27.8% vs. 9.2%; *p* = 0.037). In addition, high-flow oxygen therapy was required more frequently in patients with RSV infection (27.8% vs. 6.9%; *p* = 0.015). Allergic rhinitis was more prevalent in patients with RV infection than in those with RSV infection (53.1% vs. 5.6%; *p* < 0.001). Aeroallergen sensitization was also higher in the RV group (52.3% vs. 11.1%; *p* = 0.002), with pollen and pet dander sensitization being significantly more frequent among patients with RV infection. RV infections were most common in autumn but were detected across all seasons, whereas RSV infections predominantly occurred in winter (*p* < 0.001). WBC count, neutrophil count, eosinophil count, eosinophil percentage, and total IgE levels were significantly higher in patients with RV infection than in those with RSV infection (Table 2). A sensitivity analysis excluding children younger than 2 years yielded similar results for most RV–RSV comparisons (Supplementary Table S1). Severe exacerbation and high-flow nasal cannula use remained numerically more frequent in the RSV group, but these differences were no longer statistically significant.

A comparison of patients with single infection (*n* = 203) and coinfection (*n* = 32) among those with an identified pathogen (*n* = 235) is presented in Table 3. Among the 32 patients with coinfection, 21 (65.6%) had viral–viral combinations and 11 (34.4%) had viral–bacterial combinations; no bacterial–bacterial coinfections were identified. Allergic rhinitis (*p* = 0.014) and pollen sensitization (*p* = 0.020) were more frequently observed in patients with single infection than in those with coinfection.

**Figure 2:**
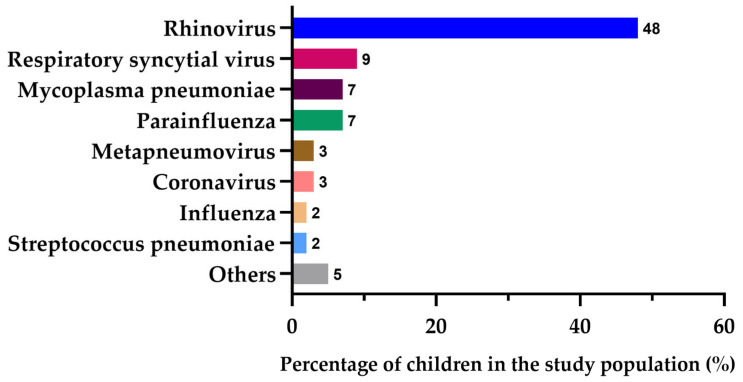
Distribution of detected respiratory pathogens in the study population. Percentages were calculated using the total study population as the denominator (*n* = 312). Pathogen categories are not mutually exclusive due to coinfections. The figure presents only detected pathogens.

The distribution of pathogens identified in the study population is shown in Figure 2. RV accounted for the majority of detected pathogens, followed by RSV, *Mycoplasma pneumoniae*, and parainfluenza virus, while other pathogens comprised a smaller proportion.

**Figure 3:**
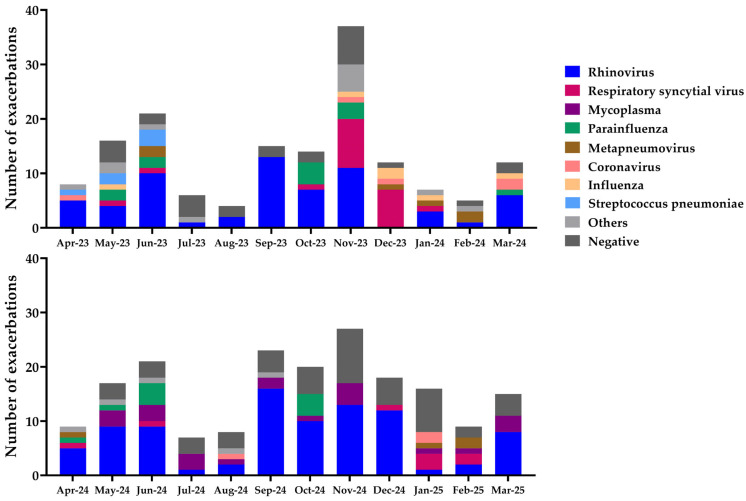
Seasonal distribution of hospitalizations and identified pathogens during the two-year study period (patient-level analysis, *n* = 312).

The seasonal distribution of hospitalizations and identified pathogens during the two-year study period is shown in Figure 3. Hospitalizations were most frequent in autumn, followed by winter. Consistent with the findings presented in Table 2, RV infections were observed throughout all seasons, with a peak in autumn, whereas RSV infections were most common in winter. *Mycoplasma pneumoniae* was detected across almost all months during the second year.

**Figure 4:**
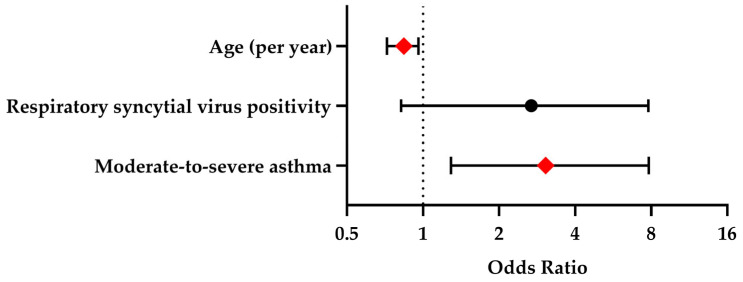
Forest plot of factors associated with severe exacerbation based on Firth penalized logistic regression. Points indicate odds ratios and horizontal lines indicate 95% confidence intervals, and the dashed vertical line indicates the null effect line (OR = 1).

Univariable logistic regression analyses for factors associated with severe exacerbation are presented in Table 4. Because only 24 severe exacerbation events were observed, multivariable associations were evaluated using Firth penalized logistic regression. The results of the multivariable Firth penalized logistic regression model are presented in Table 5. The final model included age, asthma severity, and RSV positivity. Increasing age was associated with a reduced risk of severe exacerbation (OR, 0.84; 95% CI, 0.72–0.96; *p* = 0.008), whereas moderate-to-severe asthma was independently associated with increased odds of severe exacerbation (OR, 3.06; 95% CI, 1.29–7.83; *p* = 0.011). RSV positivity was associated with higher odds of severe exacerbation (OR, 2.68), but this association was not statistically significant after adjustment (95% CI, 0.82–7.80; *p* = 0.099). The overall model was statistically significant (likelihood ratio test, *p* = 0.001), and showed acceptable discrimination (AUC, 0.71). The results are illustrated in Figure 4.

## 4. Discussion

In this two-year retrospective study of children hospitalized for asthma exacerbations, respiratory pathogens were detected in three-quarters of patients, with RV being the predominant pathogen. RV-associated exacerbations were characterized by a stronger atopic profile, including higher frequencies of allergic rhinitis, aeroallergen sensitization, eosinophilia, and elevated total IgE. RSV was detected mainly in younger children and was associated with greater severity only in descriptive comparisons. Younger age and moderate-to-severe asthma were independently associated with severe exacerbation.

RV was the most frequently detected pathogen in approximately half of the study population. Although a wide range of respiratory viruses have been associated with asthma exacerbations, RV is recognized as one of the most frequently detected viruses in childhood asthma exacerbations, likely reflecting its high prevalence among circulating respiratory viruses [11,20,21,22]. RV infections can present with a broad clinical spectrum, ranging from asymptomatic cases to severe illness requiring hospitalization [23]. However, not all RV infections in children with asthma result in exacerbations, and several factors may increase this risk, particularly atopic predisposition [13,14,24].

The frequency of allergic rhinitis and aeroallergen sensitization was higher in patients with RV infection than in those with RSV infection. In addition, eosinophil count and percentage, as well as total IgE levels, were higher in the RV group. Consistent with these findings, previous studies have demonstrated that children with aeroallergen sensitization and elevated total IgE levels are at increased risk of RV-induced asthma exacerbations [17,25,26]. In contrast, allergic sensitization does not appear to be a significant risk factor for RSV-induced wheezing in children [14,27]. Moreover, several studies have demonstrated an association between atopy and RV-induced wheezing [14,16,28,29,30,31,32]. While it is not fully established whether allergic sensitization precedes viral infection or vice versa, a clear association between allergic sensitization and RV-induced wheezing has been consistently observed [13,32].

In addition to eosinophilic inflammation, neutrophilia was observed in patients with RV infection in our study. Zheng et al. [20] similarly reported higher neutrophil levels in RV-positive patients. These findings suggest that RV-associated asthma exacerbations may be accompanied by a broader inflammatory profile characterized by both eosinophilic and neutrophilic responses. Previous studies have reported that respiratory viral infections may increase airway neutrophils and mononuclear cells during the acute phase and that impaired early antiviral responses may be associated with enhanced airway inflammation [16]. In addition, neutrophil extracellular trap formation (NETosis) and altered interferon responses have been proposed as potential mechanisms involved in RV-associated airway inflammation [33]. However, these mechanisms were not directly evaluated in our study and therefore should be considered possible explanations derived from the existing literature rather than findings demonstrated in our cohort. The coexistence of elevated eosinophil and neutrophil levels observed in patients with RV detection is consistent with these previously proposed mechanisms, although no causal inference can be made from our data.

Patients without an identified pathogen had a significantly higher median age. Consistent with this, Heymann et al. [17] demonstrated a gradual decline in pathogen detection rates with increasing age in children with wheezing. This may be attributed to narrower airways and greater susceptibility to infections in younger children, as well as more mature immune responses and an increasing role of non-infectious factors, such as aeroallergen sensitization, with advancing age. Additionally, the median age was significantly lower in patients with RSV infection compared to those with RV infection, supporting previous reports that RV infections are more common in older children, whereas RSV predominantly affects younger age groups [25,26,34].

RSV infections were most frequently observed during the winter season, followed by autumn, whereas RV infections occurred throughout the year, with a peak in autumn. This seasonal distribution is consistent with observations in the literature showing that RV circulates year-round with peaks in early autumn and late spring, while RSV is predominantly observed during the winter months [14]. Findings from the two-year study period further support these well-established epidemiological patterns.

Allergic rhinitis and aeroallergen sensitization—particularly pollen sensitization—were more frequently observed in patients with single infections compared to those with coinfections. However, no significant differences were found between the groups regarding other demographic, clinical, or laboratory parameters, including asthma severity and exacerbation severity. These findings suggest that coinfection was not associated with greater exacerbation severity in our cohort. These findings are in line with those reported by Abe et al. [25], who similarly did not observe a significant effect of coinfection on clinical severity. Although patients with coinfection were younger in our cohort, this difference did not reach statistical significance. Consistent with this trend, Abe et al. [25] reported younger age in the coinfection group, although their findings reached statistical significance, possibly due to differences in study design, including the inclusion of patients aged ≥6 months. In their study, aeroallergen sensitization was assessed using mite-specific IgE alone and was found to be more frequent in patients with single infections. In our cohort, pollen sensitization was significantly higher in the single infection group, while mite sensitization was also more frequent but did not reach statistical significance (*p* = 0.066).

Mild asthma was more common in patients with RSV infection than in those with RV infection; however, the frequency of severe exacerbations and the need for high-flow nasal cannula were higher in the RSV group in descriptive comparisons. Nevertheless, RSV positivity was not independently associated with severe exacerbation in the multivariable Firth penalized logistic regression model, whereas younger age and moderate-to-severe asthma were independently associated with severe exacerbation. In the study by Selmanoğlu et al. [35], RV and RSV were detected at relatively similar frequencies in children admitted to the intensive care unit. Although RV is generally more prevalent in the literature [6] and was also the predominant pathogen in our cohort, the higher frequency of severe clinical features among RSV-positive children in descriptive comparisons suggests that RSV detection may be associated with a more severe clinical presentation in some hospitalized children. However, this interpretation should be made cautiously because the RSV subgroup was small and RSV positivity did not remain statistically significant after adjustment. In addition, only 24 severe exacerbation events were observed in the overall cohort. Therefore, the final multivariable model was restricted to three clinically relevant variables, corresponding to an events-per-variable ratio of 8, and Firth penalized logistic regression was used to reduce small-sample bias and improve estimate stability.

This study has several limitations, including its retrospective design, absence of a control group, lack of RV subtyping, absence of viral load data, and lack of detailed information on symptom duration before sampling or recent infection history. In addition, detailed exclusion reasons were not available for all non-included patients in the initial screening population because the source database included hospitalized children who underwent respiratory pathogen testing for a broad range of clinical indications. SARS-CoV-2 was not included in the respiratory pathogen RT-PCR panel, and testing was performed only in patients with clinical suspicion of COVID-19; therefore, asymptomatic or mild SARS-CoV-2 infections may have been missed. Furthermore, because this was a single-center study conducted in a pediatric allergy and immunology clinic and included only children with documented asthma follow-up, the generalizability of the findings to all hospitalized children with wheezing or asthma-like illness may be limited. Moreover, pathogen detection was based on RT-PCR identification from nasopharyngeal samples, and no formal clinical adjudication criteria were available to determine causality. Therefore, detected pathogens, particularly bacterial organisms such as *Streptococcus pneumoniae* and *Haemophilus influenzae*, may have represented upper airway colonization rather than causative pathogens of asthma exacerbation. The number of severe exacerbation events was also relatively small (*n* = 24), resulting in an events-per-variable ratio of 8 in the final three-variable model, which limited the number of variables that could be included in the multivariable model and reduced the precision of some estimates. To mitigate small-sample bias, we used Firth penalized logistic regression; nevertheless, the confidence interval for RSV positivity remained wide and the association did not reach statistical significance after adjustment. Therefore, the independent contribution of RSV detection to exacerbation severity should be interpreted with caution and confirmed in larger cohorts. The unequal subgroup sizes, particularly the small number of children with RSV detection, should also be considered when interpreting the RV–RSV comparison, as the limited RSV sample size may have reduced statistical power and affected the precision of between-group estimates. Nevertheless, the inclusion of hospitalized children during the two-year study period, together with the evaluation of pathogen distribution, clinical characteristics, and risk factors for severe exacerbation, represents an important strength of the study.

## 5. Conclusions

Respiratory pathogens were detected in most children hospitalized for asthma exacerbation, with RV being the predominant pathogen across the two-year study period. RV detection was associated with atopic characteristics, whereas RSV detection was observed mainly in younger children. Younger age and moderate-to-severe asthma were independent predictors of severe exacerbation. These findings suggest that pathogen-related clinical patterns may provide additional clinical context, while patient age and underlying asthma severity remain important indicators of severe exacerbation.

## Figures and Tables

**Table 1:** Comparison of patient characteristics according to pathogen detection status (*n* = 312).

Characteristic	Total (*n* = 312)	Pathogen (+) (*n* = 235)	Pathogen (−) (*n* = 77)	*p* Value
Female, *n* (%)	135 (43.3)	101 (43.0)	34 (44.2)	0.856
Age, median (IQR), years	5.70 (3.42–8.79)	5.50 (3.14–8.43)	6.87 (3.90–9.90)	**0.018**
Age group, *n* (%)				0.055
≤5 years	169 (54.2)	136 (57.9)	33 (42.9)	
6–11 years	105 (33.6)	71 (30.2)	34 (44.1)	
≥12 years	38 (12.2)	28 (11.9)	10 (13.0)	
Preterm birth, *n* (%)	32 (10.3)	25 (10.6)	7 (9.1)	0.863
Family history of atopy, *n* (%)	89 (28.5)	64 (27.2)	25 (32.5)	0.377
Exposure to cigarette smoking, *n* (%)	198 (63.5)	153 (65.1)	45 (58.4)	0.292
Parental consanguinity, *n* (%)	39 (12.5)	31 (13.2)	8 (10.4)	0.655
Asthma severity, *n* (%)				0.700
Mild	168 (53.8)	128 (54.5)	40 (51.9)	
Moderate–severe	144 (46.2)	107 (45.5)	37 (48.1)	
Comorbid allergic diseases, *n* (%)				
Allergic rhinitis	143 (45.8)	102 (43.4)	41 (53.2)	0.132
Atopic dermatitis	57 (18.3)	43 (18.3)	14 (18.2)	0.982
Food allergy	19 (6.1)	13 (5.5)	6 (7.8)	0.582
Urticaria/angioedema	12 (3.8)	9 (3.8)	3 (3.9)	0.979
Anaphylaxis	5 (1.6)	4 (1.7)	1 (1.3)	0.807
Aeroallergen sensitization, *n* (%)				
Pollen	95 (30.4)	66 (28.1)	29 (37.7)	0.113
House dust mite	75 (24.0)	56 (23.8)	19 (24.7)	0.880
Pet dander	82 (26.3)	63 (26.8)	19 (24.7)	0.826
Mold	32 (10.3)	26 (11.1)	6 (7.8)	0.545
Length of hospital stay, median (IQR), days	5 (4–6)	5 (4–6)	5 (4–6)	0.709
Season, *n* (%)				0.633
Autumn	127 (40.7)	97 (41.3)	30 (38.9)	
Winter	61 (19.6)	44 (18.7)	17 (22.1)	
Spring	65 (20.8)	52 (22.1)	13 (16.9)	
Summer	59 (18.9)	42 (17.9)	17 (22.1)	
Oxygen saturation, median (IQR), %	92 (90–94)	92 (90–93)	92 (90–94)	**0.045**
PASS attack severity, *n* (%)				0.232
Mild–moderate	288 (92.3)	214 (91.1)	74 (96.1)	
Severe	24 (7.7)	21 (8.9)	3 (3.9)	
Duration of systemic corticosteroid therapy, median (IQR), days	4 (3–5)	4 (3–5)	4 (3–5)	0.992
High-flow nasal cannula, *n* (%)	24 (7.7)	19 (8.1)	5 (6.5)	0.835
WBC, median (IQR), ×10^9^/L	12.1 (9.1–15.5)	12.4 (9.2–15.9)	11.4 (8.4–15.0)	0.191
Eosinophil count, median (IQR), ×10^9^/L	0.28 (0.10–0.52)	0.26 (0.09–0.52)	0.30 (0.11–0.51)	0.426
Eosinophil percentage, median (IQR), %	2.4 (0.9–4.3)	2.2 (0.8–4.3)	2.6 (1.1–4.8)	0.203
CRP, median (IQR), mg/L	12 (5–30)	11.5 (5–30)	12 (4–32)	0.913
Total IgE, median (IQR), IU/mL (*n* = 260)	118.7 (34.3–333.3)	102.5 (33.9–307.9)	163.1 (35.1–447.8)	0.137

Abbreviations: CRP, C-reactive protein; IQR, interquartile range; PASS, Pediatric Asthma Severity Score; WBC, white blood cell count. Note: Analyses were performed at the patient level using only the first hospitalization of each child during the study period. Bold values indicate statistically significant *p* values (*p* < 0.05).

**Table 2:** Comparison of patients with single RV and RSV infections (*n* = 148).

Characteristic	RV (*n* = 130)	RSV (*n* = 18)	*p* Value
Female, *n* (%)	53 (40.8)	9 (50.0)	0.457
Age, median (IQR), years	5.8 (3.6–9.7)	3.7 (1.9–5.5)	**0.006**
Age group, *n* (%)			**0.010**
≤5 years	71 (54.6)	16 (88.9)	
6–11 years	41 (31.5)	1 (5.6)	
≥12 years	18 (13.8)	1 (5.6)	
Preterm birth, *n* (%)	13 (10.0)	1 (5.6)	0.862
Family history of atopy, *n* (%)	33 (25.4)	7 (38.9)	0.260
Exposure to cigarette smoking, *n* (%)	87 (66.9)	11 (61.1)	0.824
Parental consanguinity, *n* (%)	15 (11.5)	1 (5.6)	0.694
Asthma severity, *n* (%)			**0.044**
Mild	61 (46.9)	13 (72.2)	
Moderate–severe	69 (53.1)	5 (27.8)	
Comorbid allergic diseases, *n* (%)			
Allergic rhinitis	69 (53.1)	1 (5.6)	**<0.001**
Atopic dermatitis	20 (15.4)	2 (11.1)	0.901
Food allergy	6 (4.6)	2 (11.1)	0.251
Urticaria/angioedema	3 (2.3)	0	1.000
Anaphylaxis	3 (2.3)	0	1.000
Aeroallergen sensitization, *n* (%)	68 (52.3)	2 (11.1)	**0.002**
Pollen	42 (32.3)	1 (5.6)	**0.039**
House dust mite	42 (32.3)	2 (11.1)	0.117
Pet dander	42 (32.3)	1 (5.6)	**0.039**
Mold	18 (13.8)	0	0.130
Length of hospital stay, median (IQR), days	4 (3–6)	5.5 (3–7)	0.290
Season, *n* (%)			**<0.001**
Autumn	64 (49.2)	7 (38.9)	
Winter	17 (13.1)	11 (61.1)	
Spring	30 (23.1)	0	
Summer	19 (14.6)	0	
Oxygen saturation, median (IQR), %	91 (90–93)	91 (88–93)	0.901
PASS attack severity, *n* (%)			**0.037**
Mild–moderate	118 (90.8)	13 (72.2)	
Severe	12 (9.2)	5 (27.8)	
Duration of systemic corticosteroid therapy, median (IQR), days	4 (3–5)	4 (3–7)	0.623
High-flow nasal cannula, *n* (%)	9 (6.9)	5 (27.8)	**0.015**
WBC, median (IQR), ×10^9^/L	14.4 (11.3–17.0)	9.1 (7.9–13.3)	**<0.001**
Neutrophil count, median (IQR), ×10^9^/L	10.1 (7.8–13.3)	5.9 (3.8–8.4)	**<0.001**
Lymphocyte count, median (IQR), ×10^9^/L	2.3 (1.5–3.4)	2.3 (1.4–4.0)	0.639
Eosinophil count, median (IQR), ×10^9^/L	0.38 (0.19–0.61)	0.08 (0.02–0.24)	**<0.001**
Eosinophil percentage, median (IQR), %	2.8 (1.4–4.6)	1.1 (0.3–2.5)	**0.002**
CRP, median (IQR), mg/L	10 (5–19.3)	18 (10.5–39)	0.051
Total IgE, median (IQR), IU/mL (RV *n* = 109, RSV *n* = 16)	116.6 (43.4–350.3)	41.9 (16.9–113.9)	**0.003**

Abbreviations: CRP, C-reactive protein; IQR, interquartile range; PASS, Pediatric Asthma Severity Score; RSV, respiratory syncytial virus; RV, rhinovirus; WBC, white blood cell count. Note: Analyses were performed at the patient level using only the first hospitalization of each child during the study period. Bold values indicate statistically significant p values (*p* < 0.05).

**Table 3:** Comparison of patients with single infection and coinfection (*n* = 235).

Characteristic	Single Infection (*n* = 203)	Coinfection (*n* = 32)	*p* Value
Female, *n* (%)	90 (44.3)	11 (34.3)	0.387
Age, median (IQR), years	5.64 (3.27–8.54)	5.03 (2.48–6.96)	0.349
Age group, *n* (%)			0.786
≤5 years	116 (57.1)	20 (62.5)	
6–11 years	63 (31.0)	8 (25.0)	
≥12 years	24 (11.9)	4 (12.5)	
Preterm birth, *n* (%)	22 (10.8)	3 (9.4)	0.803
Family history of atopy, *n* (%)	58 (28.6)	6 (18.8)	0.344
Exposure to cigarette smoking, *n* (%)	134 (66.0)	19 (59.4)	0.594
Parental consanguinity, *n* (%)	26 (12.8)	5 (15.6)	0.586
Asthma severity, *n* (%)			0.241
Mild	107 (52.7)	21 (65.6)	
Moderate–severe	96 (47.3)	11 (34.4)	
Comorbid allergic diseases, *n* (%)			
Allergic rhinitis	95 (46.8)	7 (21.9)	**0.014**
Atopic dermatitis	33 (16.3)	10 (31.3)	0.073
Food allergy	10 (4.9)	3 (9.4)	0.394
Urticaria/angioedema	8 (3.9)	1 (3.1)	0.823
Anaphylaxis	4 (2.0)	0	0.423
Aeroallergen sensitization, *n* (%)	94 (46.3)	9 (28.1)	0.083
Pollen	63 (31.3)	3 (9.4)	**0.020**
House dust mite	53 (26.1)	3 (9.4)	0.066
Pet dander	56 (27.6)	7 (21.9)	0.643
Mold	24 (11.8)	2 (6.3)	0.545
Length of hospital stay, median (IQR), days	5 (4–6)	5 (4–7)	0.168
Season, *n* (%)			0.351
Autumn	88 (43.3)	9 (28.1)	
Winter	38 (18.7)	6 (18.8)	
Spring	42 (20.7)	10 (31.3)	
Summer	35 (17.2)	7 (21.9)	
Oxygen saturation, median (IQR), %	91 (90–93)	92 (90–94)	0.302
PASS attack severity, *n* (%)			0.747
Mild–moderate	184 (90.6)	30 (93.8)	
Severe	19 (9.4)	2 (6.3)	
Duration of systemic corticosteroid therapy, median (IQR), days	4 (3–5)	3.5 (3–6)	0.878
High-flow nasal cannula, *n* (%)	16 (7.9)	3 (9.4)	0.730
WBC, median (IQR), ×10^9^/L	12.6 (9.2–16.0)	11.6 (9.3–14.3)	0.380
Eosinophil count, median (IQR), ×10^9^/L	0.28 (0.10–0.53)	0.17 (0.04–0.40)	0.112
Eosinophil percentage, median (IQR), %	2.4 (0.9–4.3)	1.5 (0.3–2.9)	0.098
CRP, median (IQR), mg/L	11.5 (5.0–25.8)	13.1 (4.5–57.8)	0.511
Total IgE, median (IQR), IU/mL (single infection *n* = 175, coinfection *n* = 23)	100.5 (33.0–311.0)	124.9 (34.2–282.0)	0.931

Abbreviations: CRP, C-reactive protein; IQR, interquartile range; PASS, Pediatric Asthma Severity Score; WBC, white blood cell count. Note: Analyses were performed at the patient level using only the first hospitalization of each child during the study period. Bold values indicate statistically significant *p* values (*p* < 0.05).

**Table 4:** Univariable logistic regression analysis of factors associated with severe exacerbation (*n* = 312).

Variable	OR	95% CI	*p* Value
Age, years	0.84	0.73–0.96	0.012
Female sex	1.12	0.49–2.58	0.792
Preterm birth	0.78	0.18–3.49	0.747
Family history of atopy	1.03	0.41–2.59	0.942
Exposure to cigarette smoking	1.44	0.58–3.57	0.437
Moderate-to-severe asthma	2.50	1.04–6.03	0.041
Allergic rhinitis	0.57	0.24–1.37	0.206
Atopic dermatitis	0.89	0.29–2.70	0.833
Food allergy	0.65	0.08–5.11	0.684
Urticaria/angioedema	2.53	0.52–12.26	0.250
Anaphylaxis	3.09	0.33–28.77	0.322
Aeroallergen sensitization	0.69	0.29–1.63	0.396
House dust mite sensitization	1.33	0.53–3.35	0.542
Pollen sensitization	0.43	0.14–1.30	0.136
Mold sensitization	1.28	0.36–4.54	0.707
Pet dander sensitization	0.54	0.18–1.63	0.272
Any pathogen detection	2.42	0.70–8.35	0.162
Rhinovirus positivity	1.54	0.66–3.59	0.314
RSV positivity	3.03	1.04–8.87	0.043
Coinfection	0.78	0.18–3.49	0.747

Abbreviations: CI, confidence interval; OR, odds ratio; RSV, respiratory syncytial virus.

**Table 5:** Multivariable Firth penalized logistic regression analysis for severe exacerbation (*n* = 312).

Variable	Adjusted OR	95% CI	*p* Value
Age, years	0.84	0.72–0.96	0.008
Moderate-to-severe asthma	3.06	1.29–7.83	0.011
RSV positivity	2.68	0.82–7.80	0.099

Abbreviations: CI, confidence interval; OR, odds ratio; RSV, respiratory syncytial virus.

## Data Availability

The data presented in this study are available on request from the corresponding author. The data are not publicly available due to privacy or ethical restrictions.

## Supplementary Materials

The following supporting information can be downloaded at: https://www.mdpi.com/article/10.3390/children13070853/s1, Table S1: Comparison of patients with single rhinovirus and respiratory syncytial virus infections after exclusion of children younger than 2 years (sensitivity analysis).

## Abbreviations

The following abbreviations are used in this manuscript:

CI	Confidence interval
CRP	C-reactive protein
GINA	Global Initiative for Asthma
IQR	Interquartile range
OR	Odds ratio
PASS	Pediatric Asthma Severity Score
RT-PCR	Real-time polymerase chain reaction
RSV	Respiratory syncytial virus
RV	Rhinovirus
SPT	Skin prick test
WBC	White blood cell count
