# Supplementary material for: Respiratory Pathogens and Clinical Characteristics of Asthma Exacerbations in Hospitalized Children at a Tertiary Hospital in Türkiye
Source: Children (Basel). 2026 Jun 25;13(7):853. doi: 10.3390/children13070853 (PMC13406579; doi:10.3390/children13070853)
Supplement: Supplementary file 1 [file children-13-00853-s001.zip › children-4343969-supplementary.pdf]

Supplementary Table S1. Comparison of patients with single rhinovirus and respiratory syncytial virus infections after exclusion of children younger than 2 years (sensitivity analysis).

**Characteristic**	**RV (*n* = 115)**	**RSV (*n* = 14)**	***p* Value**
Female, *n* (%)	44 (38.3)	6 (42.9)	0.739
Age, median (IQR), years	6.0 (3.9-10.0)	3.9 (3.3-5.7)	**0.013**
Age group, *n* (%)			**0.031**
≤5 years	56 (48.7)	12 (85.7)	
6-11 years	41 (35.7)	1 (7.1)	
≥12 years	18 (15.7)	1 (7.1)	
Preterm birth, *n* (%)	10 (8.7)	1 (7.1)	0.844
Family history of atopy, *n* (%)	30 (26.1)	6 (42.9)	0.212
Exposure to cigarette smoking, *n* (%)	77 (67.0)	7 (50.0)	0.241
Consanguineous marriage, *n* (%)	11 (9.6)	0	0.608
Asthma severity, *n* (%)			**0.025**
Mild	54 (47.0)	11 (78.6)	
Moderate–severe	61 (53.0)	3 (21.4)	
Comorbid allergic diseases, *n* (%)			
Allergic rhinitis	68 (59.1)	1 (7.1)	**<0.001**
Atopic dermatitis	17 (14.8)	1 (7.1)	0.691
Food allergy	4 (3.5)	1 (7.1)	0.442
Urticaria/angioedema	3 (2.6)	0	1.000
Anaphylaxis	3 (2.3)	0	1.000
Aeroallergen sensitization, *n* (%)	66 (57.4)	2 (14.3)	**0.002**
Pollen	42 (36.5)	1 (7.1)	**0.034**
House dust mite	40 (34.8)	2 (14.3)	0.144
Pet dander	42 (36.5)	1 (7.1)	**0.034**
Mold	18 (15.7)	0	0.216
Length of hospital stay, median (IQR), days	4 (3-6)	4.5 (3-7)	0.734
Season, *n* (%)			**<0.001**
Autumn	57 (49.6)	5 (35.7)	
Winter	12 (10.4)	9 (64.3)	
Spring	28 (24.3)	0	
Summer	18 (15.7)	0	
Oxygen saturation, median (IQR), %	91 (90-93)	91.5 (89-93)	0.663
PASS attack severity, *n* (%)			0.124
Mild–moderate	106 (92.2)	11 (78.6)	
Severe	9 (7.8)	3 (21.4)	
Duration of systemic corticosteroid therapy, median (IQR), days	4 (3-5)	4 (3-5)	0.837
High-flow nasal cannula, *n* (%)	6 (5.2)	3 (21.4)	0.058
WBC, median (IQR), $\times 10^9$/L	14.0 (11.3-17.0)	8.6 (6.4-12.0)	**<0.001**
Neutrophil count, median (IQR), $\times 10^9$/L	12.3 (7.9-13.1)	5.9 (2.5-8.9)	**<0.001**
Lymphocyte count, median (IQR), $\times 10^9$/L	2.2 (1.5-3.1)	1.9 (1.3-3.1)	0.583
Eosinophil count, median (IQR), $\times 10^9$/L	0.40 (0.18-0.60)	0.09 (0.04-0.21)	**<0.001**
Eosinophil percentage, median (IQR), %	3.0 (1.4-4.6)	1.5 (0.4-2.4)	**0.009**
CRP, median (IQR), mg/L	11 (6-21)	22 (13-49)	0.042
Total IgE, median (IQR), IU/mL (*n* = 108)	182 (52-407)	56 (21-136)	**0.015**

Abbreviations: CRP, C-reactive protein; IQR, interquartile range; PASS, Pediatric Asthma Severity Score; RSV, respiratory syncytial virus; RV, rhinovirus; WBC, white blood cell count. Bold values indicate statistically significant p values ($p < 0.05$).
